# Comparative proteomic analysis reveals drug resistance of *Staphylococcus xylosus* ATCC700404 under tylosin stress

**DOI:** 10.1186/s12917-019-1959-9

**Published:** 2019-07-02

**Authors:** Xin Liu, Jinpeng Wang, Mo Chen, Ruixiang Che, Wenya Ding, Fei Yu, Yonghui Zhou, Wenqiang Cui, Xing Xiaoxu, Bello-Onaghise God’spower, Yanhua Li

**Affiliations:** College of Veterinary Medicine, Northeast Agricultural University, 600 Road Changjiang, Xiangfang, Harbin, Heilongjiang 150030 People’s Republic of China

**Keywords:** *Staphylococcus xylosus*, iTRAQ, Tylosin, Drug-resistance, Ribosomal protein L23, Thioredoxin, Aldehyde dehydrogenase a;

## Abstract

**Background:**

As a kind of opportunist pathogen, *Staphylococcus xylosus (S. xylosus)* can cause mastitis. Antibiotics are widely used for treating infected animals and tylosin is a member of such group. Thus, the continuous use of antibiotics in dairy livestock enterprise will go a long way in increasing tylosin resistance. However, the mechanism of tylosin-resistant *S. xylosus* is not clear. Here, isobaric tag for relative and absolute quantitation (iTRAQ)-based quantitative proteomics methods was used to find resistance-related proteins.

**Results:**

We compared the differential expression of *S. xylosus* in response to tylosin stress by iTRAQ. A total of 155 proteins (59 up-regulated, 96 down-regulated) with the fold-change of >1.2 or <0.8 (*p* value ≤0.05) were observed between the *S. xylosus* treated with 1/2 MIC (0.25 μg/mL) tylosin and the untreated *S. xylosus*. Bioinformatic analysis revealed that these proteins play important roles in stress-response and transcription. Then, in order to verify the relationship between the above changed proteins and mechanism of tylosin-resistant *S. xylosus*, we induced the tylosin-resistant *S. xylosus*, and performed quantitative PCR analysis to verify the changes in the transcription proteins and the stress-response proteins in tylosin-resistant *S. xylosus* at the mRNA level*.* The data displayed that ribosomal protein L23 (*rplw*), thioredoxin(*trxA*) and Aldehyde dehydrogenase A(*aldA-1*) are up-regulated in the tylosin-resistant *S. xylosus*, compared with the tylosin-sensitive strains.

**Conclusion:**

Our findings demonstrate the important of stress-response and transcription in the tylosin resistance of *S. xylosus* and provide an insight into the prevention of this resistance, which would aid in finding new medicines .

**Electronic supplementary material:**

The online version of this article (10.1186/s12917-019-1959-9) contains supplementary material, which is available to authorized users.

## Background

*Staphylococcus xylosus* (*S. xylosus*) is one of the leading causal pathogenic agent of subclinical mastitis in dairy cattle. It belongs to the coagulase-negative Staphylococcus (CNS) group. Dairy cow mastitis has resulted [1] in significant economic losses in the dairy industry worldwide [2] and this trend will continue in an exacerbating manner if urgent steps are not taken to control the activities of *S. xylosus*. Generally, macrolides and lincosamides are common antibiotics to treat infected cows [3]. Among these, tylosin is the most frequently used [4]. When bacteria are under drug seletion pressure for long time, they will evolve into resistance [5]. Thus, the long-term pressure of tylosin may lead to the prevalence of tylosin-tolerant *S.xylosus*.. Meanwhile, *S. xylosus* isolated from milk and some types of cheese exhibited multidrug resistance [6]. This has caused a series of public health problems and attracted the attention of veterinarians and dairy farmers. So, it is necessary to clearly understand the mechanisms of antibiotic resistance in order to enahnce the potency of commonly used antibiotics, and it is also helpful to find new target medicine. [7].

In general, classical antibiotic resistance mechanisms include four modes of action [8, 9]. Firstly, hydrolysis or modification leads to inactivation of drugs [8, 9]. Next, the drug target in the bacteria is altered or bypassed [8, 9]. Again, permeability changes of bacteria cell wall limit antimicrobial access. Finally, efflux exclude the antibiotic from the bacteria cell [8, 9]. In addition to the above general antibiotic resistance mechanism, there is another mechanism of acquired antimicrobial resistance, namely oxidative stress protective mechanisms which has been widely researched. Oxidative stress is a state of imbalance in the normal redox condition of cells. It occurs in cells when there is an increased production of ROS in cells without a resultant detoxification by the cell’s antioxidant defense mechanisms. This phenomenon is a major cause of apoptosis in the cells of living organisms [10]. Data from a resent investigation revealed that oxidative stress mediated pathways are responsible for the development of antibiotic resistance in bacteria [11]. Accumulating evidence have also revealed that antibiotics elicit the antibacterial effect through the initiation of oxidative stress in bacterial cells [12]. However, resistant bacteria have been reported to adapt and resist antibiotic-mediated oxidative stress by evolving a series of defensive mechanisms. They do this either through the detoxification of enzyme [13] as well as the detoxification of scavenging free radical substrates, or through their a system of DNA and protein regeneration or they are involved in competing for substrates which favor their survival and growth.

With recent advancement in proteomics, researchers have been to explore the mechanism of antibiotic resistance in bacteria. This include studies to evaluate the differences in the expression of bacterial whole protein in cells grown under different culture media or antibiotics-induced stress conditions. [14]. currently, the application of comparative proteomic methods have given researchers better understanding and insight in elucidating the mechanism behind antibiotic resistance in bacteria. Comparative proteomic methods is used as a tool to elucidate the pathways and network involved in protein regulation [15]. A very good example, is daptomycin, it was reported that LiaH protein is highly induced in cells treated with daptomycin [16]. A more recent report has shown that the level of daptomycin- resistant phenotype expressed by a bacteria is dependent on the of LiaH protein expression [17]. At the same time, quantitative proteomics method was used to monitor some important mechanisms of antibiotic resistance in clinic. [7]. It can be inferred that quantitative proteomic analysis can be used as an effective tool to reveal novel mechanism of antibiotic resistance. [7]. However, variations or alterations in the proteomics profile of *S. xylosus* against tylosin have not been reported.

This study applied the procedure of iTRAQ labeling-based quantitative proteomics to make comparison among the differentially expressed proteins of *S. xylosus* ATCC700704 with and without tolysin treatment. Bioinformatics analysis showed that some proteins could relate to transcription and stress-response, and the mechanism of tylosin resistance may be related to oxidative stress and transcription proteins.

In order to identify whether the above proteins were relevant to tylosin-resistance, *S. xylosus* ATCC700704 was induced under tylosin pressure in vitro. Quantitative PCR (qPCR) was further used to verify the candidate proteins at the mRNA level. To the best of our knowledge, this is the first study to use high-throughput labeling-based mass spectrometry (MS) to demonstrate the mechanism underlying the occurrence of tylosin resistance in *S. xylosus.* The results will provide an important insight in elucidating the mechanism of tylosin-resistant *S. xylosus*, and eliminating drug-resistant *S. xylosus* through screening the inhibitors of resistant proteins.

## Results

### Proteomics analysis of the differential expression of *S. xylosus* against tylosin

**Fig. 1:**
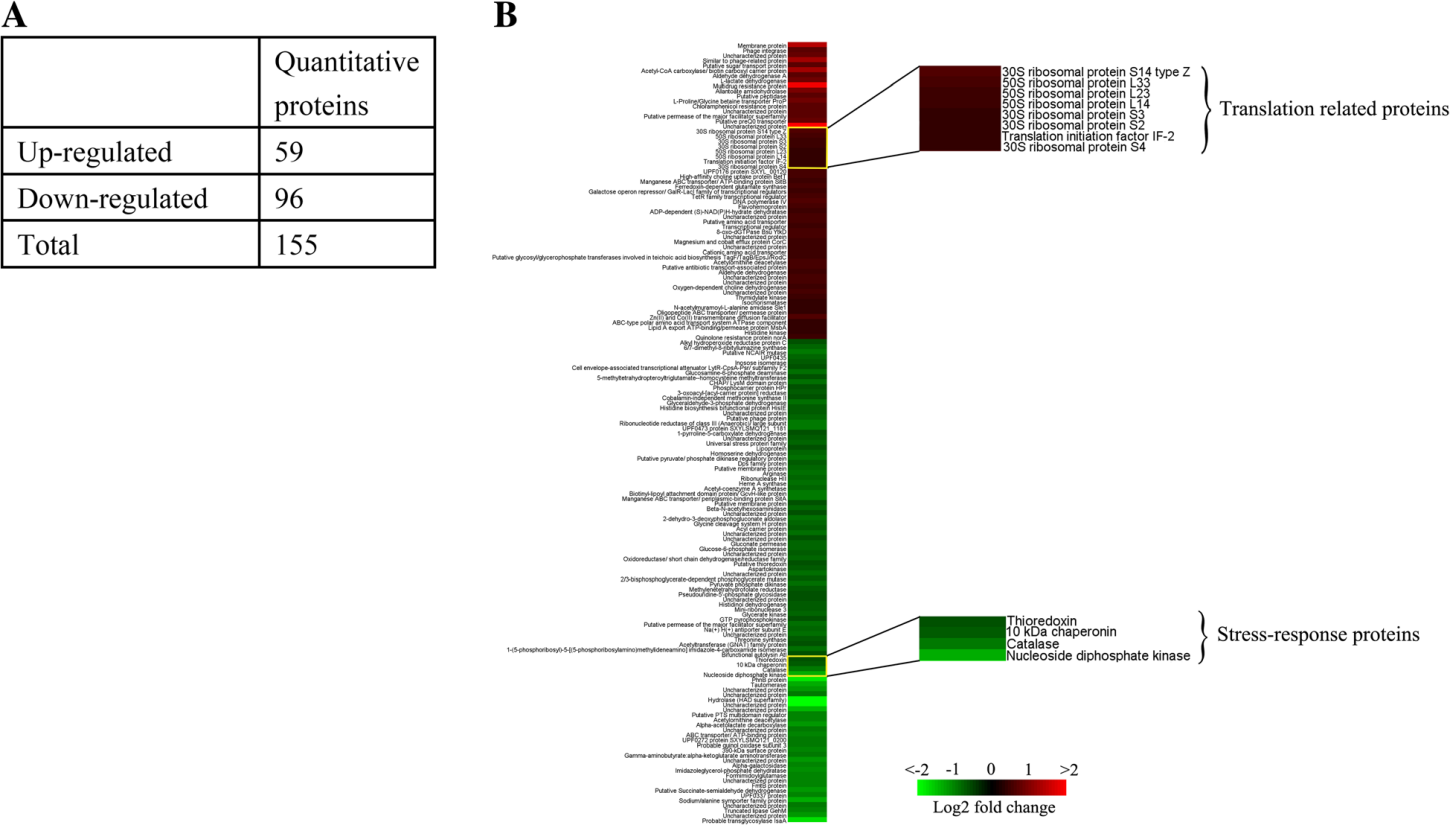
Significantly differential proteins of *Staphylococcus xylosus* ATCC700404 in 0.25 μg/mL tylosin stress using iTRAQ. **a** The number of altered proteins. **b** K-means clustering representation of total 155 DEP profiles. The magnitude of the percentage is represented by a color scale (top right) going from low (green) to high (red)

We investigated the effects of 1/2 MIC tylosin (0.25 μg/mL) on *S. xylosus* between the treated (using tylosin) and the untreated bacterial culture with isobaric reagents by quantitative proteomic analysis. And then, we pooled the samples together and then the mixture was fractioned by SCX chromatography, separated by LC and analyzed by MS/MS. Consequently, 1765 proteins were detected (Additional file 1). Based on a fold-change of >1.2 or < 0.8(with *p* value set at <0.05), 155 proteins significantly displayed differential expression between the *S. xylosus* treated with tylosin or untreated. 59 proteins were up-regulated, and 96 proteins were down-regulated (Fig. 1a). Detailed information can be found in Additional file 2 We also identified significant changes between two groups by K-means clustering heatmaps (Fig. 1b).

### Go annotation and KEGG pathway analyzed the altered proteins in *S. xylosus* under tyolsin stress

**Fig. 2:**
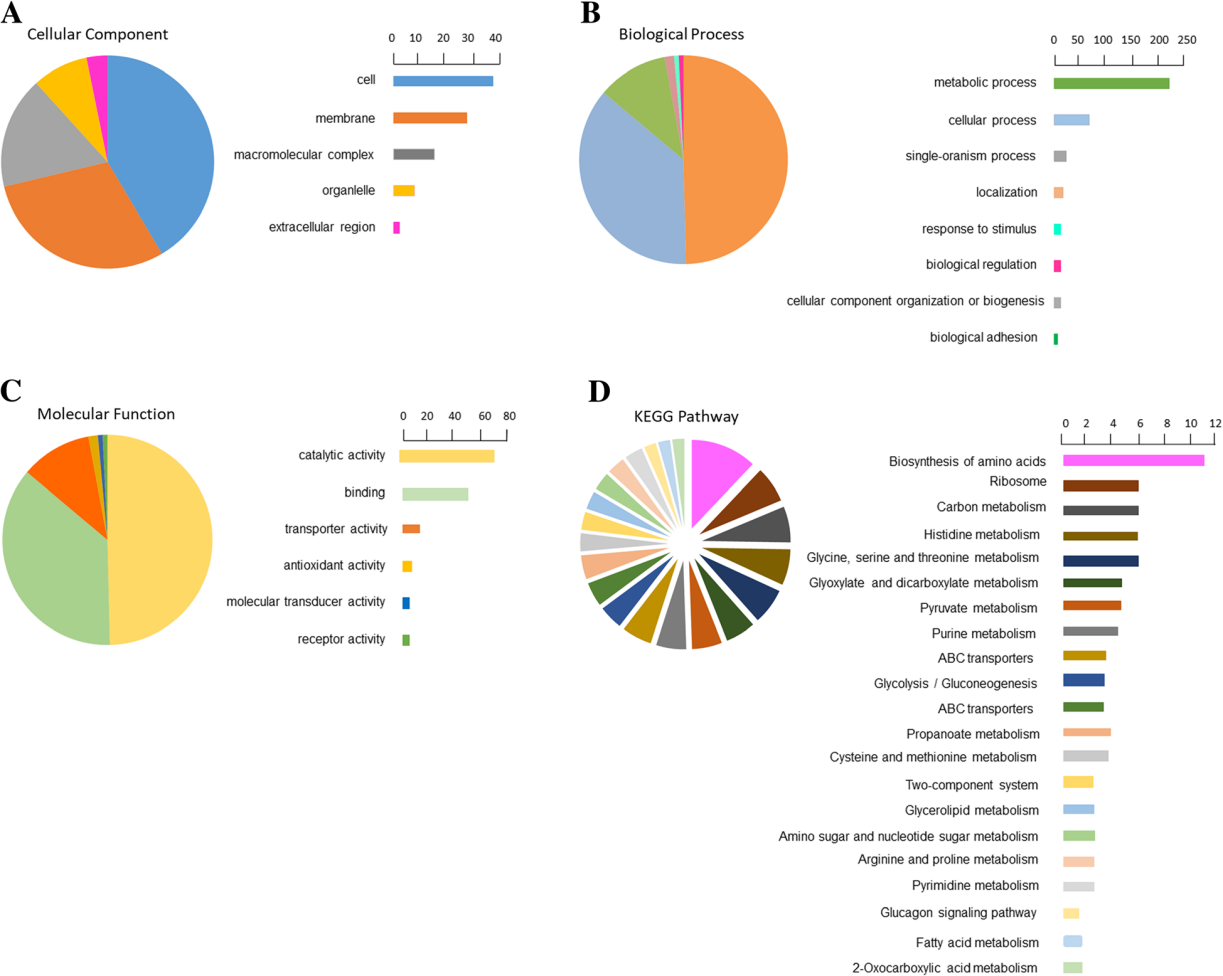
Go annotation and KEGG pathway of DEPs: Geneontology terms for subcellular laocation distribution. **a** cellular component (**b**) biological process (**c**) molecular function (**d**) main KEGG pathway

Bioinformatics were used to analyze the functional clusters of changed proteins in *S. xylosus* under tyolsin stress. Go ontology enrichment was applied to classify proteins in terms of their involvements into three main categories (biological process, molecular function and cellular component) and a specific term was used to describe each protein. Cellular component was mainly occupied by cell, membrane and macromolecular, which described the actions of a gene product at the molecular level (Fig. 2a). According to the biological process classifications, most of the proteins were associated with metabolic and cellular processes (Fig. 2b). Meanwhile, in the molecular function category, proteins related with catalytic activity and binding occupied the largest parts (Fig. 2c).

In addition, KEGG pathway analysis was applied to better understand the tylosin effects on bacteria. Pathway analysis can provide a comprehensive, systematic and direct understanding of cell biology, resistance mechanisms, and drug mechanisms of action. Generally, 64 KEGG pathways were mapped. Among these, 21 pathways were identified as statistically significant (*p* < 0.05), including biosynthesis of amino acids, ribosome, carbon metabolism and so on. The detailed information was presented in Fig. 2d. Biosynthesis of amino acids included the greatest number of proteins, followed by the ribosome and other metabolic pathways.

### Protein-protein interaction analysis

**Fig. 3:**
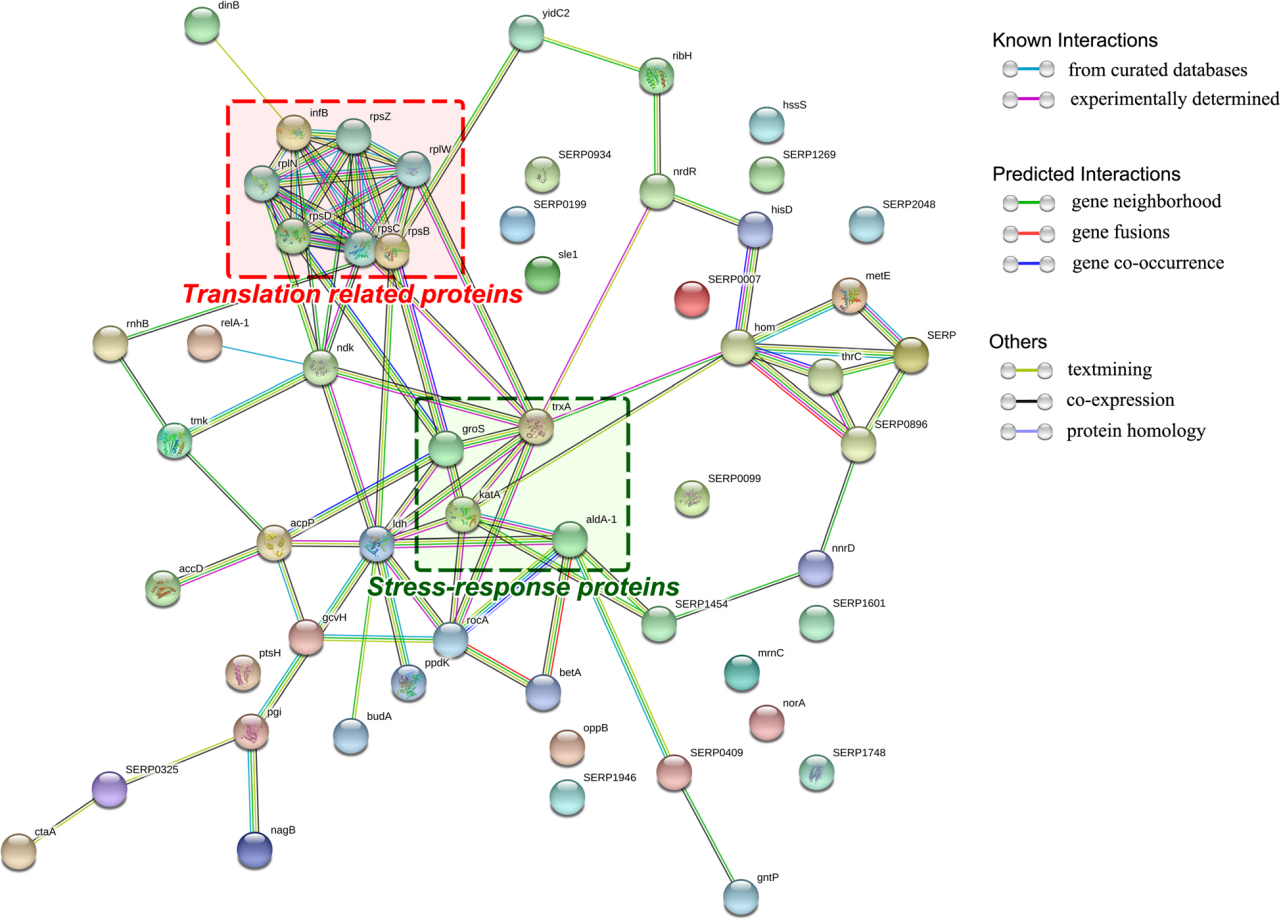
String network of significantly differential proteins of *Staphylococcus xylosus* ATCC700404 in 0.25 μg/mL tylosin stress. Colored lines between the proteins indicate the various types of interaction evidence. Structure which is drawn in the protein nodes indicated the availability of 3D protein structure information

Protein-protein interaction and network analysis were used to explain the interaction of the 155 significant differentially expressed proteins by the web-based tool STRING. The results showed that 59 proteins connected with each other in the network. Based on the STRING analysis (Fig. 3), a complicated protein-protein interaction network was identified. As shown in Fig. 3, these proteins were mainly from the two clusters, which separately constituted a related network in response to tylosin stress. One cluster was involved in translation related ribosomal subunits including 30S ribosomal protein S2 (*rpsB*), 30S ribosomal protein S4 (*rpsD*), 30S ribosomal protein S14 (*rpsZ*), 50S ribosomal protein L14 (*rplN*), 50S ribosomal protein L23(*rplW*), 50S ribosomal protein L19(*rplS*) and translation initiation factor IF-2(*infB*). Another cluster was related to stress response including chaperonin (*gros*), thioredoxin (*trxA*), aldelyde dehydrgenase (*aldA*) and catalase (*KAT*).

### Investigation of tylosin-resistant *S. xylosus* primary ribosome and stress response proteins at the mRNA level

**Fig. 4:**
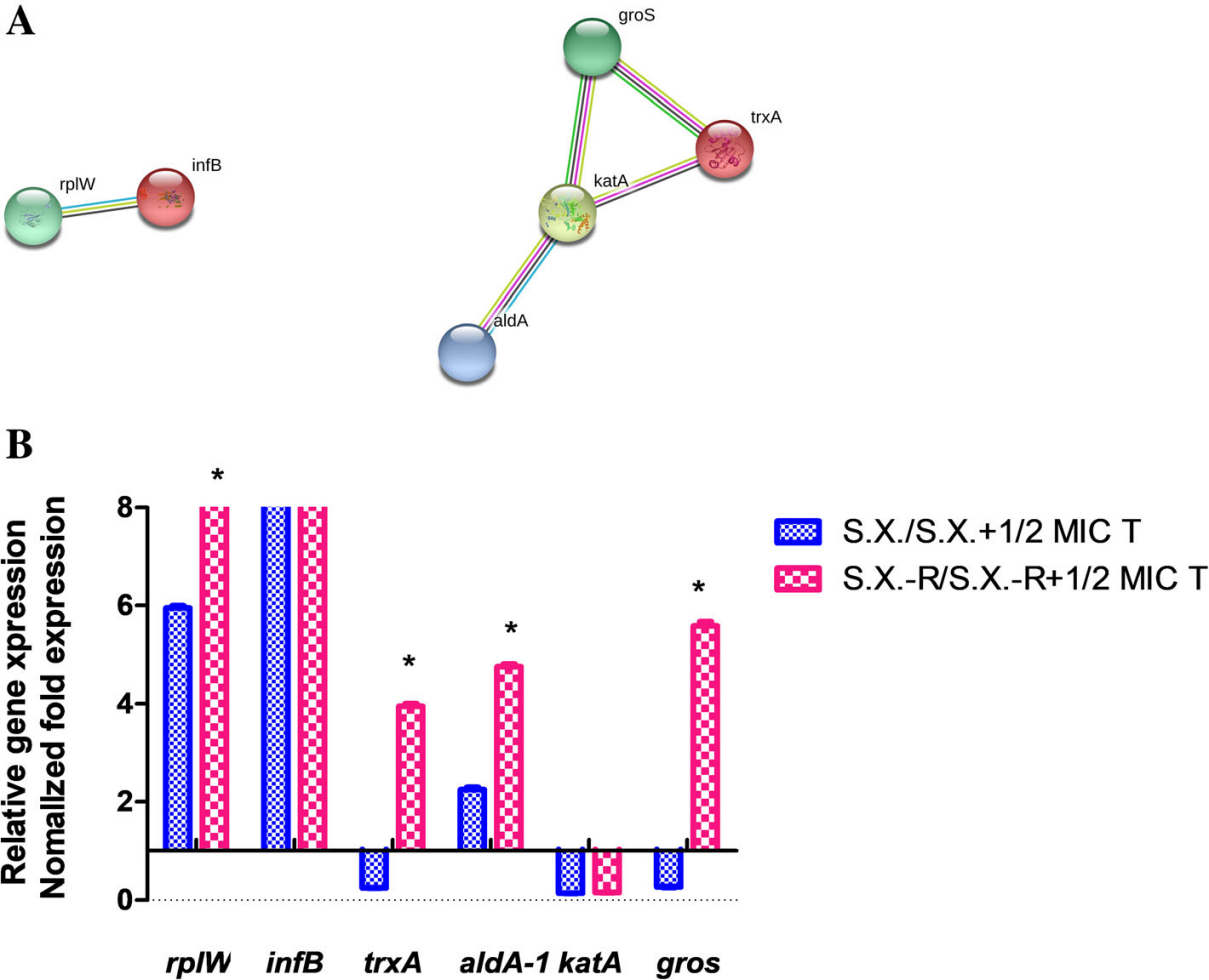
The string network and qPCR analysis of possible tylosin-resistant proteins. **a** The string network of primary tylosin-resistant proteins with altered expression. **b** The mRNA levels of two translation and four stress-response related genes were respectively analyzed by qPCR method in tylosin stress and tylosin-senstive *Staphylococcus xylosus* ATCC700404 and tylosin-resistant *Staphylococcus xylosus* ATCC700404. T: tylsoin, S.X.: tylosin-senstive *Staphylococcus xylosus* ATCC700404, S.X.-R: tylosin-resistant *Staphylococcus xylosus* ATCC700404

To identify the veracity of proteomics data, real time PCR was applied to detect the mRNA levels of six proteins in the two groups of the *S. xylosus* treated with 1/2 MIC tylosin (0.25 μg/mL) and untreated at mRNA level. Similar to the proteomics data, we observed an increase in 50S ribosomal protein L23(*rplW*), translation initiation factor IF-2(*infB*) and aldelyde dehydrgenase (*aldA*) mRNA level, whereas mRNA level of thioredoxin (*trxA*), catalase (*KAT*) and chaperonin (*gros*) decreased. In addition, in order to identify the relationship between the ribosome and stress-response with tylosin-resistance, we induced the tylosin-resistant *S. xylosus*. Then, qPCR was applied to detect the mRNA level of the proteins in tylosin-resistant *S. xylosus* (Fig. 4). The data showed that *rplW*, *trxA*, *aldA* and *gros* mRNA in tylosin-resistant *S. xylosus* was up-regulated, compared to tylosin-sensitive strains. (Fig. 4b). However, the other genes were not changed at the mRNA level.

## Discussion

Antibiotics were usually as the first line of action for veterinarians and farmers to treat infected animals even without investigating the pathogen which is the causative agent of the mastitis [18]. The prolong usage or abuse of antibiotics may lead to bacteria resistance [19, 20]. Thus, a proper understanding of the antibiotic resistance is very important to enhance the potency of most of the currently available antibiotics. And then, this will also aid in the discovery of novel targets [7]. In previous studies, resistant and mutant genes in bacteria were widely found using genomic method. [21], and so on. This method could not properly elucidate how resistant bacteria live and operate in their host systems [22]. This method was limited in scope because the procedure cannot detect biological phenotypes from the pathways of gene biosynthesis. Therefore, this method could not provide a lucid explanation of the process which led to infection [23]. However, several authors have pointed out that proteomics methods may help researchers to identify the existence of target proteins, bacterial phenotype and as well as improve the development of new therapeutic protocols [7, 24]. In the last few years, application of quantitative proteomics methods has become a powerful tool for investigation of antibiotic resistance mechanisms [25]. This is due to the complex nature of the process involved in the identification and quantification of the functional proteins associated with antibiotics treatment in bacteria.

In this study, we applied sample-pooling comparative proteomics method to investigate the differences between *S. xylosus* treated with 0.25 μg/mL tylosin and untreated. Furthermore, we carried out an in-depth study to monitor the differentially expressed proteins in the two groups to elucidate the resistant mechanism of *S. xylosus* under tylosin.

From the study, we observed that 155 proteins showed significant differences in their expression profile. This was done in line with already established criteria (FDR<1%, the folds cut off higher than 1.2 or lower than 0.8). From bioinformatics analysis, we observed that proteins such as 30S ribosomal protein S2 (*rpsB*), 30S ribosomal protein S4 (*rpsD*), 30S ribosomal protein S14 (*rpsZ*), 50S ribosomal protein L14 (*rplN*), 50S ribosomal protein L23 (*rplW*), 50S ribosomal protein L19 (*rplS*) as well as translation initiation factor IF-2(*infB*) are abundantly increased under tylosin stress. Ribosomes, the site of protein synthesis, are a major target for natural and synthetic antibiotics [26]. Among these, macrolide bind to the 50 S subunit [26–28]. Recently X-ray crystal structures revealed the molecular details of how macrolide bind to the 50 S subunit. The *Staphylococcus aureus* crystal structures of 50 S subunit with telithromycin [29–33] revealed that the drug is bound at the pocket which is in the ribosome exit tunnel, forming the typical ketolide (and macrolide) hydrogen bond between its desosamine sugar and A2058. In addition, the macrolides bind to the same site in the 50S subunit of *D. radiodurans*, at the entrance of the tunnel [26]. In our study, we utilized proteomics techniques to shed light into the mechanism of action of tylosin against *S. xylosus*. The results showed that 50 S ribosomal proteins were significantly changed, demonstrating that tylosin inhibits the *S. xylosus* by acting on 50 S subunit. On the other hand, we inferred that the reason for the increasing translation expression can be due to the fact that the 50S ribosome attacks the function of tylosin and the major role that ribosome plays in living cells [34]. The expression of target subunits for survival can be seen as a resistance strategy of *S. xylosu s*. against antibiotics. In addition, most of antibiotics inhibited the bacterial cellular energy output processes using metabolomics methods. Therefore, the upregulation of ribosomal subunits can be seen as a defensive mechanism which bacteria deploy to protect itself against the activity of tylosin. The observations in our work at the proteome level are consistent with this hypothesis.

Meanwhile, proteins relevant to stress-response, such as chaperonin (*gros*), thioredoxin (*trxA*), aldelyde dehydrgenase (*aldA*) and catalase (*KAT*) were significantly changed under tylosin stress. Generally, microorganisms induce ROS generation under various types of antibiotics, including aminoglycosides, β-Lactams and fluoroquinolones, and consequently lead to loss of cell viability [35, 36]. The oxidative stress is a state caused by the imbalance of oxidant and antioxidant levels in cells and the inability to maintain normal physiological redox-regulated regulation. [37]. Similarly, earlier reports also affirmed that the generation of ROS is very important in the mediation of toxic products in bacteria cells [38, 39] However, Thai et al. had stated that the bacteria stress response has been thought to enable bacteria survival under various natural stresses, such as heat, cold and toxicants [40]. In *Staphylococcus epidermidis*, a dysfunctional TCA cycle enables it to resist oxidative stress, leading to the *S. epidermidis* becoming less susceptible to β-lactam antibiotics [41]. At the samne time, recent study showed that antibiotic resistance include broader bacterial stress responses, such as the SOS DNA stress response, heat shock response and oxidative stress response. These antibiotic-induced responses then contribute functionally to resistance [42]. Hence, the differential expression of stress-response related proteins was a possible mechanism responsible for fighting against the tylosin.

In order to identify the relationship between the translation and stress-response related proteins with tylosin resistance in vitro*,* the tylosin-resistant *S. xylosus* was induced. Among the significantly changed proteins, 6 proteins, including 50S ribosomal protein L23 (*rplW*), translation initiation factor IF-2(*infB*), chaperonin (*gros*), thioredoxin (*trxA*), aldelyde dehydrgenase (*aldA*) and catalase (*KAT*) were selected to investigate *S. xylosus* and tylosin-resistant *S. xylosu* at the mRNA levels. Because previous studies had shown that these proteins were related to drug resistance [43–48].

As it is well known, ribosomal proteins may have been developed to improve rRNA folding and stability and could be relied upon to carry out extra ribosomal functions [49]. Regardless of the primordial sequence of events, there is increasing evidence that ribosomal proteins possess other functions apart from joining in ribosome. Several authors [49–56] have reported over 30 special functions of these group of proteins. Among these, RPL23 (*rplW*) has been identified as upregulated proteins in multidrug-resistant gastric cancer cell line and it could promote multi-resistant phenotype of gastric cancer cells [43]. In addition, Wang et al. also observed that ribosomal protein L23 mRNA was highly expressed in vincristine-resistance cells [57]. Moreover, in our study, *rplW* was found to be up-regulated in tylosin-resistant strain. Our result was in accordance with previous studies. Thus the upregulation of *rplW* is reasonable and it could be possibly connected with tylosin-resistance.

Mutations abolishing formylation of Met-tRNAi confer resistance to actinonin since bypass of the formylation step makes the deformylation step and hence the PDF activity redundant [58]. And mutations in IF2 can partially compensate for the formylation deficiency in actinonin resistant strains with a normal concentration of initiator tRNA [44]. So IF2 played important role in the resistant strains. Previously, specifically engineered mutations in domain IV can improve the activity of IF2 in initiation with non-formylated Met-tRNAi and thus increases the affinity of IF2 for Met-tRNAi [59]. Besides, the new group of IF2 mutant genes may assist in promoting the rate of initiation with Met-tRNAi by increasing the propensity of IF2 to adopt the 50S docking conformation on the 30S ribosomal subunit not only when fMet-tRNAi is present, but also in the presence of Met-tRNAi, deacylated tRNAi and elongator tRNAs. So, the IF2 was chosen to identify the relationship with the tylosin resistance. Unexpectedly, *infB(*IF2) was not significantly changed in tylosin-resistant strain. According to the above researches, the mutations in IF2 would lead to drug resistance, but not the change of expression in mRNA level. Therefore, the unchanged IF2 in tylosin resistant strain can be understood.

As technology advances, there are accumulating evidence on antibiotics resistance, thus the focus of scientists have been directed towards the development of drugs that have multiple antibacterial mechanisms, including bacterial cell wall and cellular membrane inhibition as well as the retardation or blockage of pathways involved in DNA and protein synthesis [60], while antibiotics based on inhibition of bacteria thiol-dependent redox system is a new antibiotic strategy. [61]. The thiol-dependent enzyme systems in prokaryotes are classified into two major groups and this classification is based on the presence of thioredoxin (TrxA) and glutathione (GSH) their cells [62]. One of the major distinguishing characteristics of Gram-negative and Gram-positive bacteria has to do with the presence or absence of GSH in their cells. It has been reported that almost all Gram-negative bacteria have both GSH and glutaredoxin, while Gram-positive bacteria is characterized by the presence of glutaredoxin alone [63–65]. These two systems are very significant in the survival and growth of a bacteria. They are involved in the synthesis of DNA, enhance cellular defense against oxidative stress, regenerate damaged proteins due to oxidative stress and promote post-translational modifications [62, 66]. This thiol-dependent redox systems in bacteria can serve as a potential therapeutic target in the antibacterial drug design. This was demonstrated in a recent study involving the combination of silver and ebselen as strong inhibitors of both Trx and TrxR to attenuate the occurrence MDR in Gram-negative by significantly increasing antibiotic-mediated ROS generation in treated cells [61]. From our study, the result of the mRNA level of stress-response related gene, *trxA* showed a significant increase in tylosin-resistant strain, compared with control strain. Thus, the up-regulated expression of *trxA* could fight against oxidative stress and make the strain survive under tylosin pressure.

On the other hand, when bacteria are under natural stress conditions, the generation of reactive oxygen species (ROS) would lead to DNA damage due to reactions between H_2_O_2_ and the intracellular pool of labile iron [67–69]. Dwyer et al. gave an explanation for the lethal effect of antibiotics, it was reported that cell death occurs when enough of the lesions are generated to make replication impossible to recover [70]. Meanwhile, some of the lethal effects of aminoglycosides, β-lactams, and fluoroquinolones are due to the generation of ROS [35, 36, 71]. In order to fight against the damage, aldehyde dehydrigenases metabolize aldehydes and thereby mitigate oxidative stress [46]. Aldehyde dehydrogenase (ALDH) enzymes are involved in the resistance of cancer cells to many cytostatic drugs [72–75]. Meanwhile, a recent study indicated high expression levels of ALDH enzymes in CSCs, suggesting that these molecules can cooperate in the development of drug resistance in cancers [76]. In addition, ALDHs plays important role in cell communication and signaling, regulation of gene expression, metabolic regulations, drug extrusion and drug resistance [77]. Results from this investigation revealed that *aldA* in the mRNA level was markedly upregulated, when *S. xylosus* was resistant to tylosin. Thus, *S. xylosus* may survive in tylosin pressure, via expressing aldelyde dehydrgenase to withstand oxidative stress.

Besides, bacteria can respond to threats by inducing defensive enzymes [78]. For example, overproduction of superoxide dismutase, NADH peroxidase, catalase, MutT, and MutS all reduced the killing rate of *E. coli* by classical antibiotics [45]. However, *KAT* (catalase) mRNA level was not significantly changed in tylosin-resistant strain because bacteria can oppose oxidative stress. Perhaps, defensive enzymes are not the only pathway in tylosin resistant strain to survival. In addition, previous study demonstrated that mutants in ROS scavenging enzymes—superoxide dismutase, NADH peroxidase, and catalase—may lead to bacteria resistance [79, 80]. So the mutation of catalase may exist in the tylosin-resistant strain. Finally, increased mRNA levels of chaperones were observed in the tylosin-resistant strain in this study. This is consistent with previous studies, which have found that proteins play an important role in bacterial survival, and proteins tend to unfold and aggregate to fight against stress conditions. [48]. From the foregoing, these results corroborated the findings of previous studies, and confirm that the mechanism of tylosin resistance in *S. xylosu* is complex, systemic and intricate Conclusion.

We first depicted the differentially expressed proteins of *S. xylosus* in response to tylosin stress by iTRAQ-based proteomic analysis. We observed that there are several ways against the tylosin stress in *S. xylosu*s, including ribosome and stress-response. Furthermore, we detected six related proteins in tylosin-resistant *S. xylosus* in mRNA level. The results showed that ribosomal related proteins including 50S ribosomal protein L23 (*rplW*) and stress response related proteins, thioredoxin (*trxA*), aldelyde dehydrgenase (*aldA*), catalase (*KAT*) and chaperonin (*gros*) chang significantly. So we can infer that these variations may be responsible for the tylosin resistance. Our data provided a new insight into the mechanism of tylosin resistance in tylosin-resistant *S. xylosus* and may be valuable in developing new targets in antibiotics resistant strains.

## Methods

### Cultivation of bacterial strains and determination of minimal inhibitory concentration with tylosin

*S. xylosus* ATCC700404 was grown in Tryptic Soy Broth (TSB) at 37 °C for 12 h with constant shaking [81]. Then, minimal inhibitory concentration (MIC) assay of tylosin was done as previously reported [81]. Briefly *S. xylosus* ATCC700404 was grown overnight at 37 °C.The overnight cultures were diluted in sterile physiological saline, which correspond to 1 × 10^8^ colony-forming units/mL. After that, the above cultures were diluted again with TSB till a culture concentration of 1 × 10^6^ colony-forming units/ mL was obtained. Finally, 100 mL of samples were added to a 96-well plate containing serial dilutions of tylosin in culture medium. Control bacterial culture and medium were cultivated in the absence of tylosin. The MIC was defined as the lowest concentration of tylosin to visually inhibit growth. The above assays were repeated 3 times.

### iTRAQ analysis

Protein was extracted from *S. xylosus* treated with 1/2 MIC (0.25 μg/mL) Tylosin and untreated [81]. iTRAQ analysis was conducted at Shanghai Applied Protein Technology Co., Ltd. (APT, Shanghai, China). Bioinformatics analysis was conducted as previously described [81].

#### Bioinformation

The sequence data of the differentially expressed proteins were analyzed by AgriGo gene ontology (GO) [23] mapping and annotation. In addition, the Kycto Encyclopedia of Genes and Genomes (KEGG) pathway of altered protein was further categorized utilizing the same resource. Then, the altered proteins were illustrated using K-means clustering in conjunction with a heatmap [82]. Finally, the protein-protein network of the significantly differentially expressed proteins was analyzed by STRING software.

#### Selection of tylosin-resistant mutants of *S. xylosus*

Mutants were selected by serial passage in TSB medium containing successively increasing concentration of tylosin [83].Briefly, *S. xylosus* ATCC700404(final concentration:1 × 10^6^ CFU/mL) in TSB was dispensed in a 96-well microtiter plates containing tylosin at increasing two-fold concentrations ranging from 0.5 to 16 fold which was the MIC determined for the ancestral strain. After 24 h of aerobic incubation at 37 °C, the culture containing the highest antibiotic concentration with detectable growth was used to inoculate another antibiotic dilution panel for the following passage series. The procedure was repeated until growth was obtained at a tylosin concentration of 128 μg/ml, corresponding to a 256-fold increase in antibiotic MIC for the ancestral strain. In addition, the stability of tylosin resistance was tested by serial passage (20 times) in an antibiotic-free medium. Investigation of altered proteins in the tylosin-resistant *S. xylosus* at the mRNA level was conducted by qPCR.

Total RNA was extracted from the *S. xylosus* ATCC700404 using an RNA kit according to the manufacturer^’^s instructions. Then, an equal quantity of total DNA-free RNA from the last sample was reverse-transcribed using Prim-Script TM RT reagent kit with gDNA Eraser. In this study, six proteins were chosen from the altered proteins (Table 1). Meanwhile, 16sRNA was used as an internal control and the primers used for the target genes are listed in Table 2. These reactions were performed for 40 cycles (95 °C for 15 s, 60 °C for 35 s) after initial 30s incubation at 95 °C. The assays were repeated 3 times.

**Table 1:** Proteins chosen for investigation of altered proteins in tylosin-resistant *S. xylosus*

Protein ID	Gene name	Proteins	Unique Peptides	Average iTRAQ ratio	*P*-value	Main function
A0A068E6D9	*rplW*	1	9	1.389	0.034	RPL23 is of approximately 70 proteins associated with rRNA in the large and small subunits of the ribosome, which is related to multidrug-resistant gastric cancer cell line.
C6ZDJ2	*infB*	1	1	1.366	0.044	Initiation factor (IF) 2 controls the fidelity of translation initiation,and domain III of IF2 plays a pivotal, allosteric, role in IF2 activation, which can be targeted for the development of novel antibiotics.
A0A068E9E8	*aldA-1*	2	1	1.647	0.001	The aldehyde dehydrigenases is a kind of metabolize aldehydes, thereby can mitigate oxidative stress.
A060MCA0	*trxA*	3	6	0.657	0.045	Thioredoxin play important roles in maintaining an intracellular reducing environment and combating oxidative stress in variety of organisms, including gram-positive and gram-negative bacteria.
A0A068E7D1	*katA*	4	16	0.528	0.002	The activity of catalase is used to estimate the contribution of antioxidant systems to bacterial response to oxidative stress.
A7KJI7	*gros*	1	8	0.636	0.03	The chaperones played a major role in bacterial survival under conditions of stressc.

**Table 2:** Primers for RT-PCR

Gene name	Primer Squences	Size(bp)	Annealing temp(°C)
*rplW*	F:5-TGGAAGCAAGAGACGTTCTTAAGCG-3 R:5-TAGCACGAGTATCAACGTCGAATGTG-3	99	60
*infB*	F:5-AGCAGGTGGTATTACACAGCATATCG-3 R:5-GCGTGGCCTGGCGTATCAAG-3	87	60
*trxA*	F:5-CAACTTGGTGTGGCCCTTGT-3 R:5-ACAACTTTATCTACTGGTTCGCCA-3	187	60
*aldA-1*	F:5-GTGACCGCAGGCATCGTTCC-3 R:5-CTGCTGGTATTGTTGACTCACGAATG-3	166	60
*katA*	F:5-GAGAGGTGATTCCAGAACGACGTATG-3 R:5-ATCCACGAATATCACGCTCTGCATC-3	198	60
*gros*	F: AGAGCAAACAACAAAGAGCGG R: GCCCTGCACCTACTGCAATTA	89	60
*16sRNA*	F: CGGGCAATTTGTTTAGCA R: ATTAGGTGGAGCAGGTCA	112	60

#### Statistical analysis

Values were expressed as means ± SDs. The statistical differences among the different groups were compared by 1-way ANOVA, significant means were separated using Tukey’s Honest significant difference and *p* < 0.05.

## Additional files

Additional file 1: Proteins of *S. xylosus* in response to tylosin treatment by quantitative proteomic method. Comparison between *S. xylosus* ATCC700404 and the proteins of *S. xylosus* treated with 0.25 μg/mL tylosin analyzed by iTRAQ. 1765 proteins were detected. (XLSX 207 kb)

Additional file 2: Altered proteins of *S. xylosus* in response to tylosin treatment by quantitative proteomic method. Comparison between *S. xylosus* ATCC700404 and the proteins of *S. xylosus* treated with 0.25 μg/mL tylosin analyzed by iTRAQ. Based on a fold-change of >1.2 or < 0.8(*P*-value<0.05), 155 proteins significantly displayed differential expression. (XLSX 2612 kb)

## Data Availability

The datasets generated and analyzed during the current study are available in the link “https://figshare.com/account/home”.

## Abbreviations

ALDH: Aldehyde dehydrogenase
CNS: Coagulase-negative Staphylococcus
iTRAQ: Isobaric tag for relative and absolute quantitation
KEGG: Kycto Encyclopedia of Genes and Genomes
LC-MS/MS: Iquid chromatography-tandem mass spectrometry
MS: Mass spectrometry
qPCR: Quantitative PCR
*S. xylosus*: *Staphylococcus xylosus*
